# A novel homozygous RSPH4A variant in a family with primary ciliary dyskinesia and literature review

**DOI:** 10.3389/fgene.2024.1364476

**Published:** 2024-05-16

**Authors:** Chenling Shen, Yilin Shen, Weiyi Huang, Andi Zhang, Tianyuan Zou, Dongye Guo, Hao Wang, Jichang Wu, Haixia Hu, Mingliang Xiang, Bin Ye

**Affiliations:** ^1^ Department of Otolaryngology and Head and Neck Surgery, Ruijin Hospital, Shanghai Jiao Tong University School of Medicine, Shanghai, China; ^2^ Department of Audiology and Speech-Language Pathology, College of Health Science and Technology, Shanghai Jiao Tong University School of Medicine, Shanghai, China; ^3^ Department of Otolaryngology and Head and Neck Surgery, Shanghai Children’s Hospital, Shanghai Jiao Tong University School of Medicine, Shanghai, China

**Keywords:** mixed hearing loss, novel mutation, primary ciliary dyskinesia, *RSPH4A*, whole-exome sequencing

## Abstract

**Introduction:** Primary ciliary dyskinesia (PCD) is a rare heterogeneous disease caused by abnormalities in motile cilia. In this case report, we first analyzed the clinical and genetic data of a proband who was suspected of having PCD on the basis of her clinical and radiological findings.

**Methods:** Whole-exome sequencing was performed, and a variant in the *RSPH4A* gene was identified in the proband. Sanger sequencing was used for validation of *RSPH4A* variants in the proband, her sister, her daughter and her parents. Finally, the phenotypic features of the patient were analyzed, and the current literature was reviewed to better understand the gene variants in PCD related to hearing loss and the clinical manifestations of the *RSPH4A* variant in PCD.

**Results:** The chief clinical symptoms of this proband included gradual mixed hearing loss, otitis media, anosmia, sinusitis, recurrent cough and infertility. Her DNA sequencing revealed a novel homozygous T to C transition at position 1321 within exon 3 of *RSPH4A* according to genetic testing results. This variant had never been reported before. The homozygous variant resulted in an amino acid substitution of tryptophan by arginine at position 441 (p.Trp441Arg). The same variant was also found in the proband’s sister, and a heterozygous pathogenic variant was identified among immediate family members, including the proband’s daughter and parents.

**Discussion:** A literature review showed that 16 pathogenic variants in *RSPH4A* have been reported. Hearing loss had only been observed in patients with the RSPH4A (c.921+3_6delAAGT) splice site mutation, and the specific type of hearing loss was not described.

## Introduction

Primary ciliary dyskinesia (PCD) is a rare genetic disease related to abnormal ciliary structure or function. It is usually inherited in an autosomal recessive pattern, with few X-linked recessive forms. The prevalence of PCD is estimated to be between 1:2000 and 1:40000. In children with recurrent respiratory infection, the incidence is as high as 5% (Chapelin et al., 1997). PCD is mainly caused by defects in the ciliary outer dynein arms, radial spokes (RSs) or central pair (CP), which lead to severely impaired mucociliary clearance (Edelbusch et al., 2017). The main clinical manifestations of PCD include neonatal respiratory distress, bronchiectasis, recurrent upper or lower respiratory infections, and infertility in adulthood (Goutaki et al., 2016). Kartagener’s syndrome is a subgroup of PCD, presenting with a triad of bronchiectasis, sinusitis, as well as *situs inversus*. It accounts for approximately half of patients with PCD (Kennedy et al., 2007). In pediatric patients with PCD, respiratory and otologic symptoms are common. The slow progression of PCD may affect the growth and development of children. Since detection methods require a certain level of skill, the diagnosis of PCD is usually delayed. In Europe, the median age at diagnosis of PCD is 5.3 years (Kuehni et al., 2010). In China, the median patient age at diagnosis is 7.0 years (Guan et al., 2021). Therefore, it is necessary for Chinese doctors, especially respiratory physicians, pediatricians, and ENT doctors, to improve their awareness of this disease. Eighty-one percent of patients with PCD have recurrent otitis media, including otitis media with effusion (OME) and acute or chronic otitis media, at an age of 2–3 years (Sommer et al., 2011). Recurrent ear infections and otitis media with effusion can lead to temporary or permanent hearing loss, which may lead to delayed speech development during childhood (Pruliere-Escabasse et al., 2010). More than 90% of children with hearing loss have ever been diagnosed of otitis media for at least one time due to the abnormality of mucociliary clearance in the middle ears and Eustachian tubes (Kreicher et al., 2018). In adult patients with PCD, the median age at diagnosis is 23.5 years (Shah et al., 2016). Adults have a higher prevalence of chronic wet cough, sinusitis, diffuse panbronchiolitis, *Pseudomonas aeruginosa* infection, and radiological bronchiectasis and show worse lung function (Peng et al., 2022). Therefore, if a patient’s chief symptoms are unexplained ENT manifestations, including chronic rhinitis, recurrent or chronic rhinosinusitis and recurrent otitis media, it is important for specialists to be aware of PCD. The diagnosis of PCD mainly relies on typical clinical manifestations combined with biopsy for ultrastructural examination with electron microscopy, nasal nitric oxide testing, gene detection, high-speed video microscopy and immunofluorescence (Goutaki and Shoemark, 2022). Treatments of PCD are largely referred to the international guidelines of cystic fibrosis, which focus on improving the ability of mucus clearance and anti-bacterial treatments of airway infections. Moreover, latest precision medicine therapies, such as mRNA transcription and gene therapy, have also been explored as possible treatment options. The identification of specific genotypes in PCD and their underlying mechanisms is the first step to achieving personalized medicine. The ultimate goal is to restore ciliary function (Paff et al., 2021).

More than fifty genes have been found to be associated with PCD, including *DNAH11*, *DNAH5*, *DNAI1*, *CCDC39*, and *CCC40* genes. The most frequently mutated genes reported in North America are *DNAH*5 and *DNAI1* genes, followed by *DNAH11*, *CCDC39*, and *CCDC40* genes. Pathogenic variants in these five genes account for more than 50% of PCD cases (OConnor et al., 2021). In China, the highest incidence of variants occurs in *DNAH11* gene, followed by *DNAH5*, *CCDC39*, *DNAH1*, and *CCNO* genes (Guan et al., 2021).

Here, we report a PCD patient with a novel *RSPH4A* pathogenic variant in a Chinese family. Studies have shown that *RSPH4A* localizes to the radial spoke heads of the microtubules in cilia. Variants in *RSPH4A* mainly destroy the ciliary ultrastructure and lead to the absence of radial spoke heads, which causes dysfunction of mucus clearance (Zhao et al., 2021). A range of motility defects, including reduced ciliary beat frequency, dyskinesia and rotational rather than planar beating patterns, have been reported in PCD patients with *RSPH4A* variants through nasal biopsies (Frommer et al., 2015). In China, the incidence of variants in *RSPH4A* gene reported in children with PCD is 1.33% (Guan et al., 2021). To date, a total of five adult PCD patients with *RSPH4A* variants in China have been reported by Chinese researchers (Bian et al., 2021; Wang et al., 2022; Feng et al., 2023).

## Materials and methods

### Subjects

Here we described the clinical feature of a young woman with primary ciliary dyskinesia. A three-generation family tree was drawn according to available information obtained from hospital records as well as interview of her immediate family members. The pathogenic variant was also verified on patient’s sibling, parents and daughter. This study was approved by the research ethics committee in Ruijin Hospital. Written informed consents were obtained from all participants.

### DNA purification and genetic testing

We performed whole-exome sequencing to detect the presence of any variant. Genomic DNA was extracted from peripheral blood lymphocytes using standard protocols according to previous references (Hu et al., 2022). The sequencing was performed by capturing high-throughput chip technology. The platforms for whole-exome sequencing (NanoWES Human Exome, Berry Genomics Corporation, Beijing, China) were performed on a Illumina NovaSeq 6000 (Illumina, San Diego, United States). Sanger sequencing was used to verify the variants in patients and her family members.

### *In silico* analyses

Single-nucleotide variants (SNVs) and short insertion and deletions (InDels) discovery were performed according to the American College of Medical Genetics and Genomics (ACMG) guidelines (Richards et al., 2015). High-frequency variants (minor allele frequency>0.01) were ruled out combined with using HPO (https://hpo.jax.org/app/), OMIM (https://www.ncbi.nlm.nih.gov/omim/) and GHR (https://ghr.nlm.nih.gov/genetics) database. SNVs and InDels were filtered as follows: 1) Noncoding and intronic variants were excluded; 2) Synonymous missense variants were filtered; 3) Homozygous or compound heterozygous variants were retained. No pathogenic SNV or InDel variant which had been previously reported related to the phenotype of our case was detected. However, one candidate missense variant in *RSPH4A* was found. A further detailed genetic study of 78 genes (Supplementary Table S1) was carried out by panel sequencing of causal and risk genes known to be associated with ciliary dyskinesia reported by the American College of Medical Genetics and Genomics (ACMG) (Miller et al., 2022). The functional effects and the pathogenicity of the variant were predicted and analyzed using Verita Trekker^®^ variant detection system and Enliven^®^ variant annotation interpretation system independently developed by Berry Genes (Berry Genomics Corporation, Beijing, China) (Hu et al., 2022).

### Literature review

A literature review was firstly done using Pubmed for all previously reported articles published between 1975 and 2023 which were related to hearing loss and PCD. A combination of relevant medical subject heading terms along with keywords such as: “cilia,” “ciliary,” “dyskinesia,” “immotile,” “motility,” “hearing,” “hearing loss,” “impairment,” and “deafness” were used (see details of the search strategy in Supplementary Table S2). The reference lists of the relevant articles were also reviewed. Full text articles and conference abstracts of the identified citations were retrieved and reviewed to determine their eligibility for inclusion. In addition, the keyword “*RSPH4A*” was searched using LOVD3 (Leiden Open Variation Database) (www.lovd.nl/ *RSPH4A*) and PubMed to summarize all pathogenic variants in *RSPH4A* and related clinical manifestations reported so far.

## Results

### Clinical findings

**FIGURE 1:**
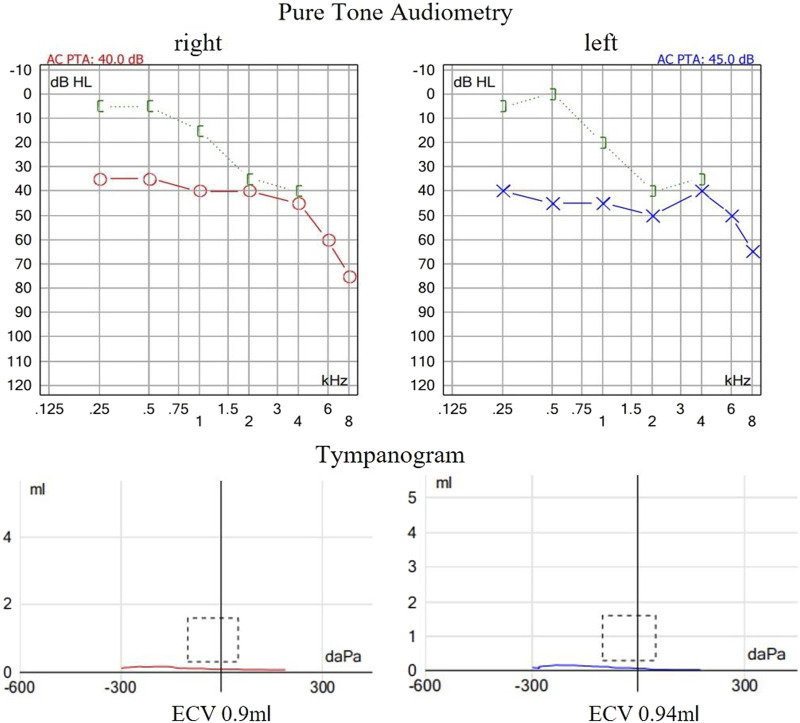
Auditory examination of the proband. The pure tone audiometry revealed bilateral conductive hearing loss at low frequencies and sensorineural hearing loss at high frequencies. Acoustic immittance showed flat tympanogram which indicated bilateral secretory otitis media.

**FIGURE 2:**
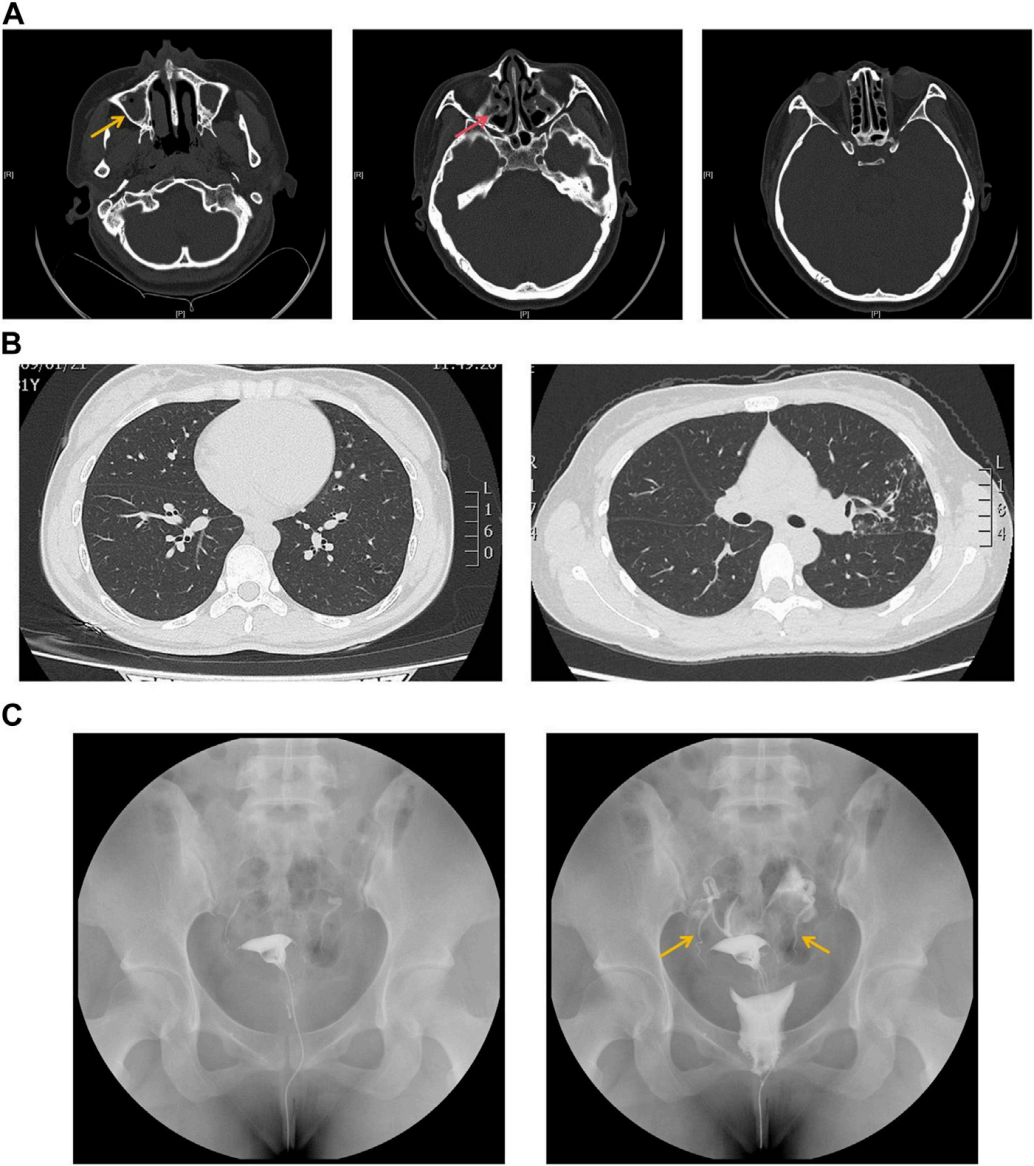
**(A)** Sinus computed tomography showed inflammation in both maxillary (yellow arrow) and ethmoidal sinuses (red arrow). **(B)** Chest computed tomography showed bronchiectasis in both sides of lung lobes. **(C)** Salpingography showed no obstruction in bilateral fallopian tubes but was not patent like as normal (yellow arrow).

The proband is a 31-year-old female patient with gradual hearing loss in the past 3 years accompanied by ear dullness (Figure 1). She also complained of hyposmia and recurrent sinusitis since childhood. Nasal steroids were used to control nasal symptoms (Figure 2A). She had a prolonged wet cough after suffering from COVID-19 and was diagnosed with bronchiectasis by chest computed tomography (Figure 2B). In addition, the patient reported infertility after marriage. Salpingography showed no obvious obstruction in the bilateral fallopian tubes, but they were not as patent as normal (Figure 2C). She finally had a daughter assisted by reproductive technology after fertility consultation. When asking the proband about her family history, she reported that her sister had similar symptoms. The proband’s parents and daughter did not show any symptoms at the time. The parents were not close relatives. No family history of primary ciliary dyskinesia had ever been identified.

### Genetic findings and *in silico* predictions

**FIGURE 3:**
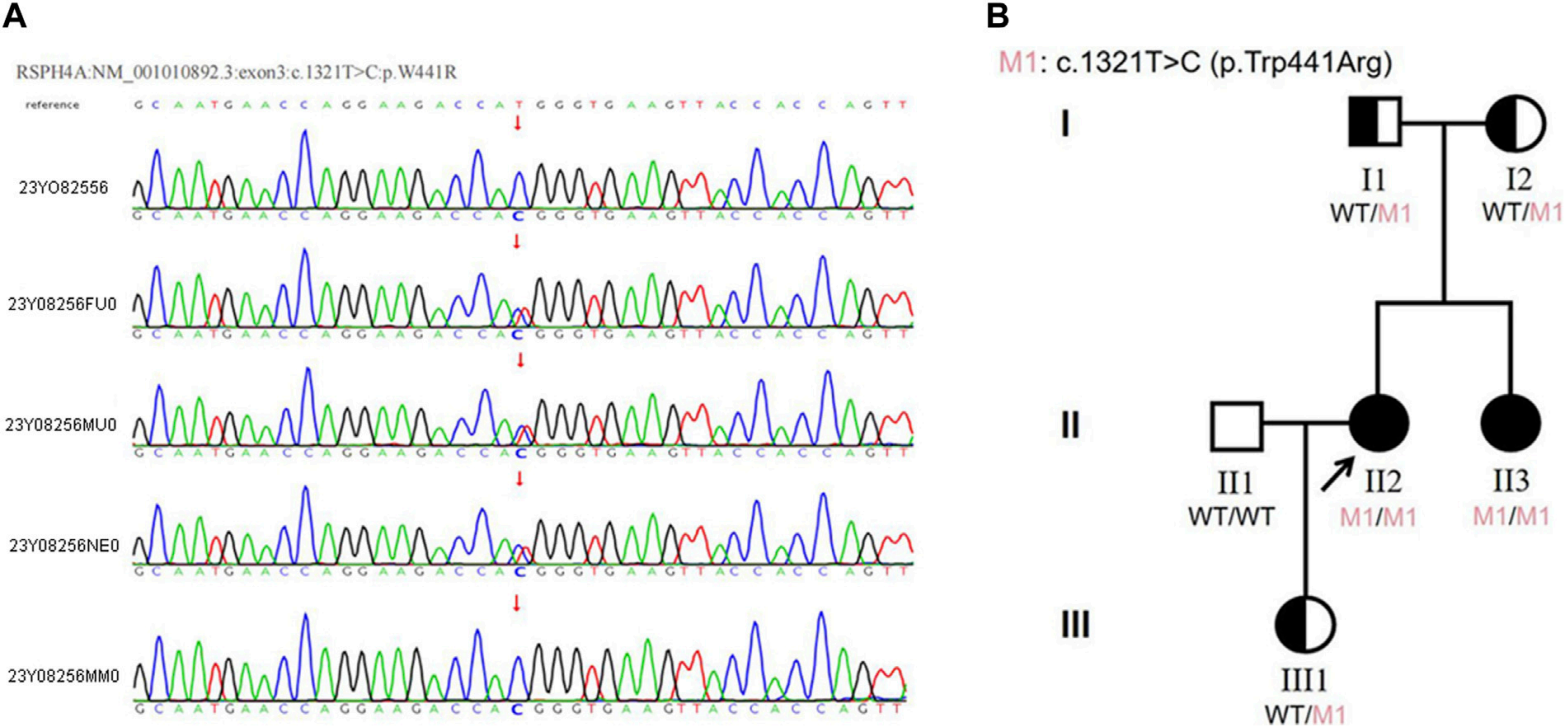
**(A)**The results of Sanger sequencing in this family. The homozygous variant in the *RSPH4A* gene was found in both the proband and her sister. The variant was found in the heterozygous state in the proband’s parents and daughter. (23Y08256: proband, 23Y08256FU0: father, 23Y08256MU0: mother, 23Y08256NE0: daughter, 23Y08256MM0: sister). **(B)** Pedigree of the proband’s family. The *RSPH4A* variant was found in the proband (II-2). The patient was diagnosed with ciliary dyskinesia at 33 years of age. Arrow indicates proband. One family member-her sister (II-3) had the same clinical symptoms and was also diagnosed with ciliary dyskinesia. Proband and her sister were homozygous for the variant in *RSPH4A*. Their parents (I1 and I2) and proband’s daughter (III1) all carried the heterozygous variant in *RSPH4A*.

We sequenced the DNA of patients using Agilent SureSelect Whole Exome capture and Illumina sequencing technology. No pathogenic SNV or InDel variant that had been reported to be related to the phenotype in our patient was detected. However, a homozygous missense variant in exon 3 of *RSPH4A* was detected. According to ACMG guidelines and recommendations from the ClinGen Sequence Variant Interpretation (SVI) working group (Biesecker et al., 2018), the variant in *RSPH4A* (NM_001010892.3:exon3:c.1321T>C:p.Trp441Arg) was considered a likely pathogenic variant (PP3+PM2+ PM3+PP1). This variant was further analyzed by REVEL (Rare Exome Variant Ensemble Learner) (Ioannidis et al., 2016), and the results showed that it could cause harmful effects. This variant has not been reported in the 1000 Genomes Project Database, Exome Aggregation Consortium (ExAC) Database or Genome Aggregation Database (gnomAD). According to public databases, *RSPH4A* (OMIM:612647) variants have been verified to cause primary ciliary dyskinesia. Therefore, this novel variant was considered likely pathogenic combined with the clinical features and family history. Sanger sequencing verified a novel T to C transition at position 1321 within exon 3 of *RSPH4A,* resulting in an amino acid substitution of tryptophan by arginine at position 441 (Trp441Arg), which led to dysfunction of the *RSPH4A* protein (Figure 3A).

### Verification of *RSPH4A* in the patients and family members

The proband reported that her sister had the same clinical manifestations. Sanger sequencing was also used to verify the *RSPH4A* variant in her family members. The homozygous variant of *RSPH4A* was also detected in the proband’sister. The variant was found in the heterozygous state in the proband’s parents and daughter (Figure 3A). A family pedigree was drawn (Figure 3B).

### Literature review of genes related to hearing loss in PCD

No review has identified genes related to hearing loss in PCD. A literature review was performed by searching the keywords, such as “cilia,” “ciliary,” “dyskinesia,” “immotile,” “motility,” “hearing,” “hearing loss,” “impairment,” and “deafness.” Our searches extracted 108 articles from Pubmed, of which 6 articles met our eligibility criteria (Supplementary Figure S1). Our literature review revealed that the hearing loss was different depending on the gene involved (Table 1). Variants in *CCDC114* cause sensorineural hearing loss (SNHL) (Li et al., 2019), while variants in *CCN*O, *CDH3*, *DNAH5* and *OFD1* cause conductive hearing loss (CHL) (Bukowy-Bieryllo et al., 2019; Fan et al., 2019; Henriques et al., 2021; Wang et al., 2021; Yang et al., 2022). In addition, the clinical findings were summarized to conclude the different symptoms, age at onset or diagnosis among PCD patients with CHL and SNHL (Table 2). The first visit to a doctor of PCD patients with CHL was always before 10 years of age, while the time of patients with SNHL was often approximately 10 years of age or later. However, the age at diagnosis was almost after 10 years, demonstrating the lack of awareness of this disease by most clinicans. According to the literature review, CHL is the most common type of hearing loss in PCD due to otitis media, acounting for about 88.9% and only one PCD patient had SNHL. All patients with CHL and SNHL had bronchiectasis and rhinosinusitis. Five patients (62.5%) with CHL also suffered from gastro-oesophageal refux and three patients (32.5%) had *situs inversus*. The patient with SNHL had the unique symptoms of pulmonary artery stenosis, renal fibrosis and patent ductus arteriosus. These findings implied that specific genotypes of PCD could have different underlying pathogenic mechanisms.

**TABLE 1:** Different genes variants and their corresponding type of hearing loss.

Gene name	Gender	Hearing loss	Age at onset of symptoms (y)	Age at diagnosis (y)	Reference
*CDH3&DNAH5*	Female	CHL^a^	9	11	Fan et al. (2019)
*CCDC114*	Female	SNHL^b^	10	15	Li et al. (2019)
*CCNO*	Female	CHL	<1	6	Henriques et al. (2021)
*DNAH5*	Female	CHL	7	1	Wang et al. (2021)
*OFD1*	Male	CHL	31	29	Yang et al. (2022)
*OFD1*	Male	CHL	<1	16	Bukowy-Bieryllo et al. (2019)
*OFD1*	Male	CHL	<1	16	
*OFD1*	Male	CHL	<1	20	
*OFD1*	Male	CHL	<1	6	

a CHL: Conductive hearing loss.

b SNHL: Sensorineural hearing loss.

**TABLE 2:** Comparison of clinical characteristics in PCD patients with CHL and SNHL.

	CHL^a^ (total n = 8) n (%)	SNHL^b^ (total n = 1) n (%)
Gender		
Male	5 (62.5)	0
Female	3 (37.5)	1 (100.0)
Age at diagnosis		
<1 year old	0	0
1–10 years old	3 (37.5)	0
	5 (62.5)	1 (100.0)
Age at onset of symptoms		
<1 year old	5 (62.5)	0
1–10 years old	2 (25.0)	1 (100.0)
	1 (12.5)	0
Medical history		
Recurrent pneumonia	2 (25.0)	0
Bronchiectasis	8 (100.0)	1 (100.0)
Rhinosinusitis	8 (100.0)	1 (100.0)
Otitis media	8 (100.0)	0
Vision loss	1 (12.5)	1 (100.0)
Pulmonary artery stenosis	0	1 (100.0)
Renal fibrosis	0	1 (100.0)
Patent ductus arteriosus	0	1 (100.0)
Chronic bronchitis	1 (12.5)	1 (100.0)
Hypotrichosis	1 (12.5)	0
Situs inversus	3 (37.5)	0
Gastro-oesophageal refux	5 (62.5)	0

a CHL: Conductive hearing loss.

b SNHL: Sensorineural hearing loss.

### Literature review of *RSPH4A* variants in PCD and their clinical symptoms

*RSPH4A* variants have rarely been reported in China. Up to January 2023, a total of 85 cases with 18 pathogenic variants in *RSPH4A* had been reported in the Leiden Open Variation Database (www.lovd.nl/ *RSPH4A*). A literature review was performed through the Litvar and PubMed databases and altogether 10 articles from 1875-2023 were selected which described the clinical manifestations of patients with altogether 18 pathogenic variants in *RSPH4A* gene in details (Supplementary Table S3). We then summarized the clinical features of patients with different varients in *RSPH4A* for comparison (Table 3). The most common features of *RSPH4A* variants in PCD patients were bronchiectasis, rhinosinusitis, otitis media, followed by infertility, recurrent pneumonia and chronic bronchitis. Hearing loss was only reported in one patient of *RSPH4A* (exon1:c.460C>T:p.Gln154) without describing the specific type (Castleman et al., 2009).

**TABLE 3:** Comparison of clinical characteristics of PCD patients with RSPH4A variants.

	Patients reported with variants in RSPH4A (total n = 18) n (%)
Medical history	
Recurrent pneumonia	5 (27.8)
Atelectasis	1 (5.6)
Bronchiectasis	17 (94.4)
Rhinosinusitis	15 (83.3)
Otitis media	11 (61.1)
Hearing loss	1 (5.6)
Chronic bronchitis	4 (22.2)
Situs inversus	0
Infertility	9 (50.0)
Deformity	3 (16.7)

## Discussion

In this study, we reported a patient with gradual hearing loss, otitis media, anosmia, sinusitis, recurrent cough and infertility. She was diagnosed with PCD after performing whole-exome sequencing. A novel homozygous in *RSPH4A* (NM_001010892.3:exon3:c.1321T>C:p.Trp441Arg) was found. This genetic variant has not been reported before in the literature on individuals with RSPH4A-related disease. Therefore, doctors should be aware of this disease, especially when young patients experience mixed hearing loss. PCD is usually inherited in an autosomal recessive pattern. The pathological basis of PCD is abnormal ciliary movement, which leads to dysfunction of mucociliary clearance in the middle ear and Eustachian tubes. Therefore, PCD patients may have auditory manifestations at an early age. Hearing loss in children will lead to speech or language delay. Therefore, it is necessary to regularly monitor hearing in children with PCD (Shapiro et al., 2016). Symptoms are often alleviated with age, although some patients continue to require hearing aids in adulthood (Lucas et al., 2020). Kreicher et al., 2018 found that CHL was the most common type of hearing loss in PCD patients, although 30% of children had some sensorineural component to their hearing loss. With the treatment of CHL caused by otitis media, hearing thresholds in PCD children can often be improved following conservative management. Tympanogram scores in different age groups also showed a trend toward improvement with age. Most cases resolve by the age of 12 (Wolter et al., 2012). Ventilation tube insertion in children with PCD is controversial because postoperative prolonged otorrhea and persistent perforation of the tympanic membrane are common complications. Hearing aids are recommended in patients with moderate and severe hearing loss (Andersen et al., 2016). In addition to the most common manifestation of secretory otitis media, sensorineural hearing loss can also be found in some PCD patients. We speculate that this may be closely related to abnormal ciliary function in the cochlear hair cells, although to date, no researcher has reported pathological structural and functional changes in hair cells of PCD patients or animal models. PCD is usually caused by motile cilia dysfunction due to variants in genes that encode axonemal motor proteins that are responsible for ciliary beat regulation. However, latest studies have shown that these proteins also play important roles in sensory function (Piatti et al., 2017). Mutation of different proteins may cause corresponding clinical features. From the above literature review, we conclude that pathogenic variants in the *CCDC114* gene may cause SNHL, while variants in *CCNO*, *CDH3*, *DNAH5*, and *OFD1* genes may cause CHL, suggesting that different types of hearing loss may be closely related to the specific location and function of different proteins. Sometimes manifestations of motor and sensor ciliopathies can overlap.

The difference in the clinical manifestations of PCD usually depends on the specific location of the mutated protein in the cilia. The cross section of cilia shows a typical “9 + 2” configuration, with 9 peripheral microtubule doublets surrounding the central microtubule pair. The outer dynein arm and inner dynein arm are attached to the microtubule doublets and provide motor activity. Radial spoke and nexin-dynein regulatory complexes ensure stability (Horani and Ferkol, 2021). PCD patients whose bronchial biopsies showed the absence of central microtubules under transmission electron microscopy had a higher incidence of otologic features and respiratory tract infections (Tamalet et al., 2001). For example, compared with patients with variants in *DNAH5* gene or other outer dynein arm defects, those with variants in *CCDC39* or *CCDC40* gene have worse lung function and growth indices (Davis et al., 2015; Davis et al., 2019). Patients with variants in *CCNO* and *MCIDAS* genes that cause a reduced number of cilia also exhibit more severe pulmonary lesions (Boon et al., 2014). Individuals with variants in genes that encode the ciliary outer dynein arm or radial spoke, including *DNAH11*, *DNAH9* and *RSPH1* genes, tend to have less severe respiratory or otologic symptoms (Knowles et al., 2014; Fassad et al., 2018; Shoemark et al., 2021). Therefore, exploring the association between the genotype and phenotype of PCD patients is helpful for the long-term management of disease and accurate prediction of prognosis.

RSPH4A localizes to the radial spoke heads and plays a central role in the three types of RS assembly. It is generally found in the trachea, ependymal tissues and oviduct of mice and modulates the planar beating of motile cilia (Yoke et al., 2020). The research team of De Jesus-Rojas first identified a pathogenic variant in *RSPH4A* gene (c.921 + 3_921+6delAAGT) in Puerto Rico in 2021 and traced it back to the ancestral haplotype. Sixty-nine percent of the patients had hearing loss, but the specific type of hearing loss was not explicitly reported. Bian et al. reported homozygous variants in the *RSPH4A* gene (c.667delA, p.S223Afs*15) in a patient with neurofibromatosis and confirmed the diagnosis of PCD at the same time. No hearing loss was found in this patient. Wang et al. reported three novel compound *RSPH4A* variants: 1) c.2T>C, p. (Met1Thr), 2) c.1774_1775del, p. (Leu592Aspfs*5), and 3) c.351dupT, p. (Pro118Serfs*2), which were related to infertility in PCD patients in China. The symptoms were similar to those reported in our case, but no hearing loss was found in these three families. Hearing was also normal in a child with a variant of c.1454G>A (p.Arg485Gln) in *RSPH4A* gene (Guan et al., 2021)*.* In the above literature review, the main clinical features of PCD caused by *RSPH4A* variants were summarized, including bronchiectasis, rhinosinusitis, otitis media and infertility. Only one article reported symptom of hearing loss caused by *RSPH4A* variant, and not all variants in *RSPH4A* would cause otitis media and hearing loss. One of the most obvious features the patient exhibited in our case was mixed hearing loss, which had never been reported before in *RSPH4A*-related PCD. Thus, we consider that different *RSPH4A* variants exert different effects on cilia movement and function.

We believe that it is important for ENT doctors not only to focus on the patient’s otologic or nasal *symptoms* but also to pay attention to the clinical manifestations in other organs and the medical history. In this case, we simply connected the patient’s hearing loss and secretory otitis media to her long-term sinusitis at first, but mixed hearing loss was found when more attention was given to the auditory examination. Then, her bronchiectasis and infertility and the similar symptoms in her sister made us further suspect a genetic disease. Finally, gene sequencing confirmed the diagnosis of PCD. Untill now, the treatments of PCD still focus on treating the symptoms and no therapy has been shown to be effective in restoring ciliary function. As a result, expanding the genetic spectrum of this disease will not only raise the awareness of PCD by doctors, but also lead to timely intervention and can put emphasis on therapeutic plan. Different variants will also promote the development of personalized gene therapy in the future.

## Data Availability

The datasets for this article are not publicly available due to concerns regarding participant/patient anonymity. Requests to access the datasets should be directed to the corresponding author.
