# Supplementary material for: A novel homozygous RSPH4A variant in a family with primary ciliary dyskinesia and literature review
Source: Front Genet. 2024 May 16;15:1364476. doi: 10.3389/fgene.2024.1364476 (PMC11137616; doi:10.3389/fgene.2024.1364476)
Supplement: Supplementary file 1 [file Table1.DOCX]

| Variant | Article | Clinical features |
| --- | --- | --- |
| Exon1, c.460C>T  （p.Gln154） | Castleman et al, 2009[30] | Reduced exercise tolerance, chronic wet cough, recurrent respiratory infections, bronchiectasis, and nasal symptoms such as rhinorrhea, rhinitis, nasal blockage, sinusitis, ear obstruction, hearing loss |
| Exon1, c.325C>T  （p.Gln109） | Ewa Ziętkiewicz, 2012[31] | Recurrent upper respiratory tract infections, recurrent pneumonia, chronic bronchitis, bronchiectasis, sinusitis and otitis media |
| Exon3, c.1068G>A  （p.Try356*） |  |  |
| Exon3, c.1468C>T  （p.Arg490*） |  |  |
| Exon1, c.116C>A  （p.Ser39） | Daniels et al, 2013[32] | Respiratory distress in term neonates, laterality (situs) defects, bronchiectasis, otitis media |
| Exon4, c.1732_1733del  （p.Asp578Argfs*3） |  |  |
| Exon1, c.462_469del  （p.Gln155ThrfsTer12） | Kott et al, 2013[33] | Bronchiectasis, sinusitis, subfertility, rhinosinusitis, otitis media, COPD |
| Exon3, c.1391G>A  （p.Gly464Glu） |  |  |
| Exon5, c.1916+2T>A  （p.?） |  |  |
| Exon3, c.1351C>T  （p.Gln451*) |  |  |
| Exon1, c.166dup  （p.Arg56Profs*11） | Casey et al, 2015[34] | Recurrent lower respiratory tract infections, wet cough |
| Exon1, c.667del  （p.Ser223Alafs*15） | Bian et al, 2021[17] | Productive cough, bronchiectasis, sinusitis, immobile sperm |
| Exon2, c.921+3_6delAAGT  （p.Tyr230Glnfs?8） | De Jesús-Rojas et al, 2021[35] | Wet cough, daily nasal congestion, neonatal respiratory distress, bronchiectasis, chronic secretory otitis media, infertility |
| Exon? c.1454G>A  （p.Arg485Gln) | Guan et al, 2021[6] | Chronic wet cough, sinusitis, bronchiectasis, thoracic deformity, atelectasis |
| Exon1,c.2T>C, p.(Met1Thr) | Wang et al, 2022[18] | Oligoasthenoteratozoospermia, infertility, sinusitis，bronchiectasis |
| Exon4, c.1774_1775del, p.(Leu592Aspfs*5) |  |  |
| Exon1,c.351dupT, p.(Pro118Serfs*2) |  |  |
| Exon?,c.194_224del, c.298delGly | Feng et al, 2023[19] | Bronchiectasis, pansinusitis, type II respiratory failure, severe malnutrition |

Supplementary Table 3. Ten ariticles of eighteen pathogenic variants in *RSPH4A* and their corresponding clinical features.
