# Supplementary figures and images for: A novel homozygous RSPH4A variant in a family with primary ciliary dyskinesia and literature review
Source: Front Genet. 2024 May 16;15:1364476. doi: 10.3389/fgene.2024.1364476 (PMC11137616; doi:10.3389/fgene.2024.1364476)

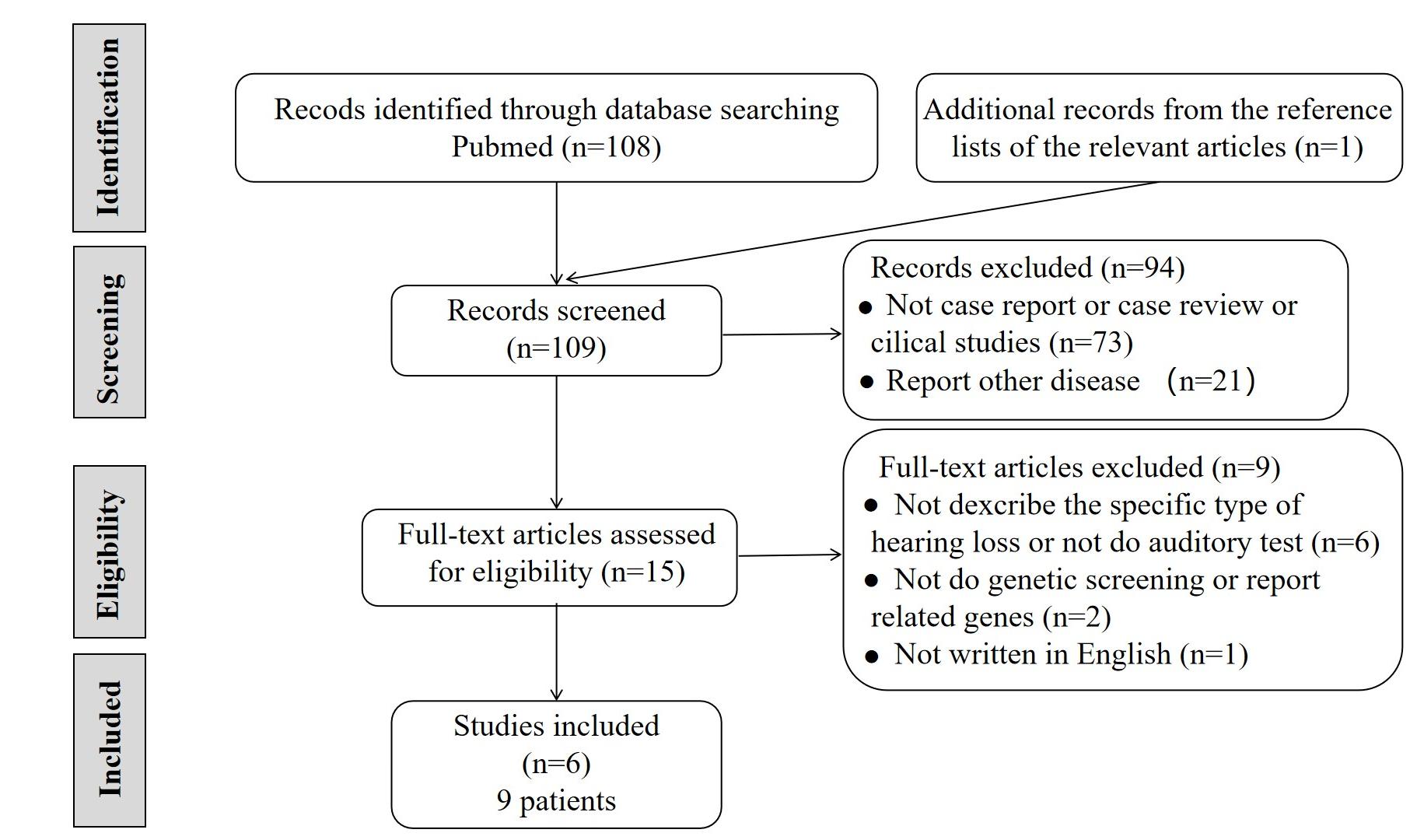

Supplement: Supplementary file 2 [file Image1.JPEG]
