# Supplementary material for: A novel homozygous RSPH4A variant in a family with primary ciliary dyskinesia and literature review
Source: Front Genet. 2024 May 16;15:1364476. doi: 10.3389/fgene.2024.1364476 (PMC11137616; doi:10.3389/fgene.2024.1364476)
Supplement: Supplementary file 3 [file Table2.DOCX]

| **No** | **Query in English** | **Results** |
| --- | --- | --- |
| #1 | ciliary motility disorders | 2,758 |
| #2 | immotile cilia syndrome | 3,021 |
| #3 | ciliary dyskinesia | 3,548 |
| #4 | primary ciliary dyskinesia | 3,452 |
| #5 | immotile cilia | 561 |
| #6 | #1 OR #2 OR #3 OR #4 OR #5 | 3,889 |
| #7 | hearing | 168,300 |
| #8 | hearing loss | 105,965 |
| #9 | hearing impairment | 112,736 |
| #10 | deafness | 47,340 |
| #11 | #7 OR #8 OR #9 OR #10 | 188,559 |
| #12 | #6 AND #11 | 111 |
|  | 1975-2023 | 108 |

Supplementary Table 2. Searching strategy of key words about PCD and hearing loss.
