# Supplementary material for: A novel homozygous RSPH4A variant in a family with primary ciliary dyskinesia and literature review
Source: Front Genet. 2024 May 16;15:1364476. doi: 10.3389/fgene.2024.1364476 (PMC11137616; doi:10.3389/fgene.2024.1364476)
Supplement: Supplementary file 4 [file Table3.DOCX]

| Seventy-eight supplementary genes related to ciliary dyskinesia | | | | | |
| --- | --- | --- | --- | --- | --- |
| *ACTA2*  *ACTC1*  *ACVRL1*  *APC*  *APOB*  *ATP7B*  *BMPR1A*  *BRCA1*  *BRCA2*  *BTD*  *CACNA1S*  *CASQ2*  *COL3A1* | *DSC2*  *DSG2*  *DSP*  *ENG*  *FBN1*  *FLNC*  *GAA*  *GLA*  *HFE*  *HNF1A*  *KCNH2*  *KCNQ1*  *LDLR* | *LMNA*  *MAX*  *MEN1*  *MLH1*  *MSH2*  *MSH6*  *MUTYH*  *MYBPC3*  *MYH11*  *MYH7*  *MYL2*  *MYL3*  *NF2* | *OTC*  *PALB2*  *PCSK9*  *PKP2*  *PMS2*  *PRKAG2*  *PTEN*  *RB1*  *RET*  *RPE65*  *RYR1*  *RYR2*  *SCN5A* | *SDHAF2*  *SDHB*  *SDHC*  *SDHD*  *SMAD3*  *SMAD4*  *STK11*  *TGFBR1*  *TGFBR2*  *TMEM127*  *TMEM43*  *TNNI3*  *TNNT2* | *TP53*  *TPM1*  *TRDN*  *TSC1*  *TSC2*  *TTN*  *VHL*  *WT1*  *BAG3*  *DES*  *RBM20*  *TNNC1*  *TTR* |

Supplementary Table 1. Supplementary 78 genes recommended by ACMG SF v3.1 which are related to ciliary dyskinesia. No pathogenic variants were found after using filters for variant type, population variant frequency, age at onset, functional impact of the variant and pathogenicity predictions using Enliven® variant annotation interpretation system.
